# The role of southern red-backed voles, *Myodes gapperi*, and *Peromyscus* mice in the enzootic maintenance of Lyme disease spirochetes in North Dakota, USA

**DOI:** 10.1016/j.ttbdis.2024.102385

**Published:** 2024-08-02

**Authors:** Michael W. Dougherty, Nathan M. Russart, Robert A. Gaultney, Emily M. Gisi, Haley M. Cooper, Lindsey R. Kallis, Catherine A. Brissette, Jefferson A. Vaughan

**Affiliations:** a Department of Biology, University of North Dakota, Grand Forks, ND, United States; b Department of Medicine, University of Florida College of Medicine, University of Florida, Gainesville, FL, United States; c Aldeveron, Fargo, ND, United States; d Department of Biomedical Sciences, North Dakota School of Medicine and Health Sciences, University of North Dakota, Grand Forks, ND, United States; e Institute of Tissue Medicine and Pathology, University of Bern, Bern, Switzerland

**Keywords:** Lyme disease, *Ixodes scapularis*, *Dermacentor variabilis*, *Peromyscus*, *Myodes gapperi*, Reservoir potential

## Abstract

Lyme disease has expanded into the Great Plains of the USA. To investigate local enzootic transmission, small mammals were trapped in two forested tracts in northeastern North Dakota during 2012 and 2013. *Peromyscus* mice and southern red-backed voles, *Myodes gapperi*, comprised over 90% of all mammals captured. One site was dominated by *Peromyscus* (79% of 100 mammals captured). At the other site, *M. gapperi* (59% of 107 mammals captured) was more abundant than *Peromyscus* (36%). Immature stages of two tick species parasitized small mammals: *Dermacentor variabilis* and *Ixodes scapularis*. Larval *I. scapularis* ectoparasitism was significantly higher on *Peromyscus* (81% infested; 3.7 larvae per infested mouse) than *M. gapperi* (47% infested; 2.6 larvae per infested vole) whereas larval and nymphal *D. variabilis* ectoparasitism were highest on *M. gapperi*. Over 45% of infested rodents were concurrently infested with both tick species. Testing engorged *I. scapularis* larvae from *Peromyscus* (*n* = 66) and *M. gapperi* (*n* = 20) yielded xenopositivity prevalence for *Borrelia burgdorferi* sensu lato (s.l.) in these rodents of 6% and 5%, respectively. Progeny of field collected *M. gapperi* were used to determine host infectivity for a local isolate of *B. burgdorferi* sensu stricto (s.s.). Five *M. gapperi* were injected with spirochetes, infested with pathogen-free *I. scapularis* larvae on days 10, 20, and 40 after infection, and engorged larvae molted to nymphs. Subsamples of nymphs were tested by PCR for *B. burgdorferi* s. s. DNA and yielded infection rates of 56% (*n* = 100 nymphs tested), 75% (*n* = 8) and 64% (*n* = 31), respectively. The remaining infected nymphs were fed on BALB/c *Mus musculus* mice and 7 d later, mice were euthanized, and tissues were cultured for *B. burgdorferi* s.s. Nymphs successfully transmitted spirochetes to 13 of 18 (72%) mice that were exposed to 1–5 infected ticks. Theoretical reservoir potentials – i.e., ability to generate *B. burgdorferi* infected nymphs – were compared between *Peromyscus* and *M. gapperi*. At one site, *Peromyscus* accounted for nearly all *Borrelia*-infected nymphs produced (reservoir potential value of 0.935). At the other site, the reservoir potentials for *Peromyscus* (0.566) and *M. gapperi* (0.434) were comparable. The difference was attributed to differences in the relative abundance of voles versus mice between sites and the higher level of ectoparasitism by larval *I. scapularis* on *Peromyscus* versus *M. gapperi* at both sites. The southern red-backed vole, *M. gapperi*, contributes to the enzootic maintenance of Lyme disease spirochetes in North Dakota and possibly other areas where this rodent species is abundant.

## 1. Introduction

Lyme disease is the most common vector-borne disease in the United States. Approximately 30,000 new cases of Lyme disease are reported annually to the US Centers for Disease Control and Prevention (CDC, 2024) but estimates based on medical insurance claims suggest that the actual number of new cases occurring annually may be higher (ca. 476, 000 cases) (Kugler et al., 2021). Most Lyme disease cases in the US occur in the Northeast and Upper Midwest regions of the country where *Borrelia burgdorferi* sensu lato (s.l.) spirochetes are transmitted to humans *via* the prolonged attachment (>24 h) of infected blacklegged ticks, *Ixodes scapularis*. Lyme disease is a zoonosis requiring pathogen transmission between the tick vector and non-human vertebrate reservoirs. In the Northeast and Upper Midwest, the white-footed mouse (*Peromyscus leucopus*), is regarded as the most important vertebrate reservoir responsible for maintaining the zoonotic transmission of Lyme disease spirochetes (Anderson et al.,1985; Levine et al.,1985; Mather et al., 1989; Halsey et al., 2018).

**Fig. 1.**
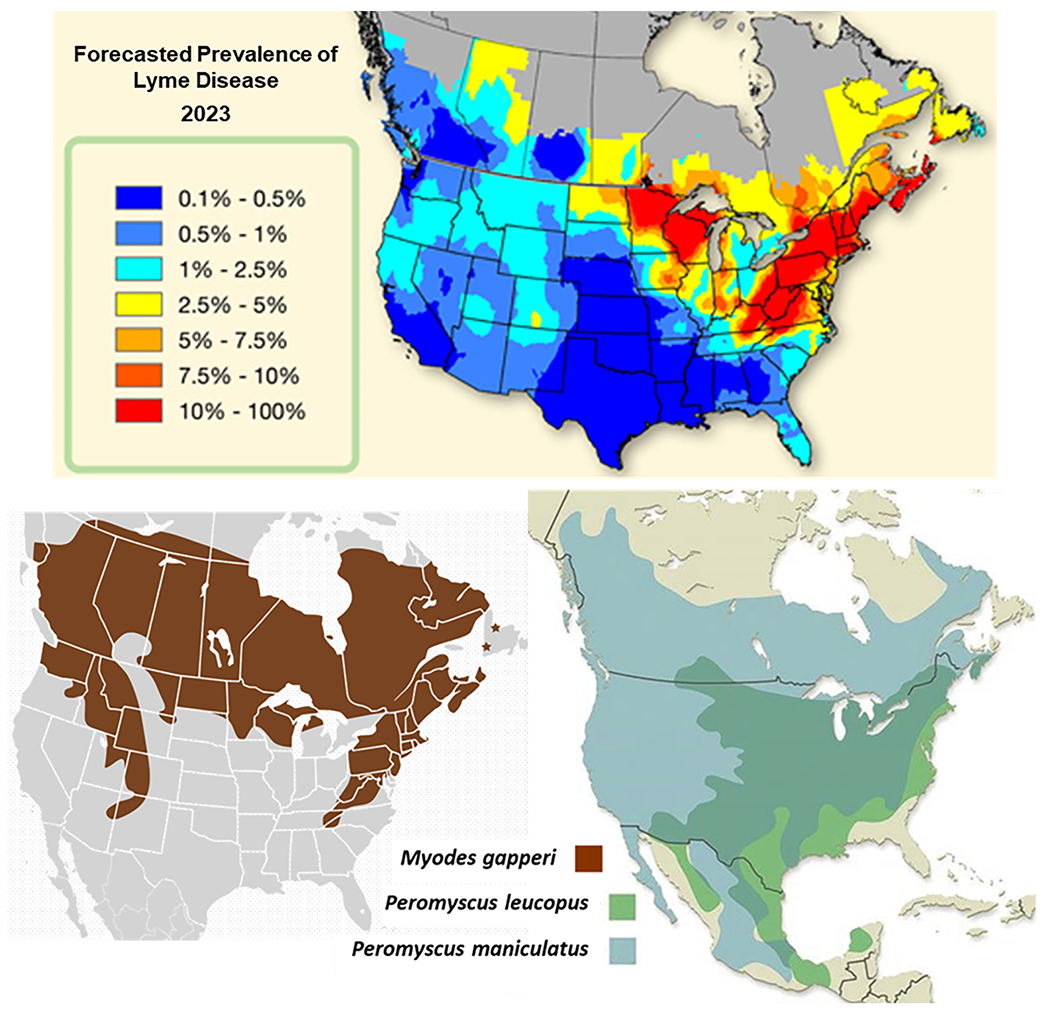
Geographic distributions of the red backed vole, *Myodes gapperi* (Naughton 2012), *Peromyscus leucopus, P. maniculatus* (Machtinger and Williams 2020) and estimated Lyme disease prevalence in companion animals (Companion Animal Parasite Council).

In recent years, the geographic range of *I. scapularis* has expanded simultaneously on two fronts – northward into Canada (Gasmi et al., 2016; Clow et al., 2017; Gabriel-Rivet et al., 2017), and westward into the Great Plains states (Maestas et al., 2016,^2018^; Oliver et al., 2017; Noden et al., 2024). As a result, the vertebrate fauna encountered by blacklegged ticks is likely to change. If *I. scapularis* continues to move northward, the predominance of *P. leucopus* is likely to give way to more northerly-distributed rodent species, such as *P. maniculatus* (=deer mouse) and *Myodes gapperi* (=southern red-backed vole) (Fig. 1). The host infectivity of *P. maniculatus* for *B. burgdorferi* s.l. has been shown to be equivalent to that of *P. leucopus* (Rand et al., 1993; Peavey and Lane, 1995). There is less known about the host infectivity of *M. gapperi* voles.

In a 2010 survey of ticks in North Dakota, Russart et al. (2014) first detected the presence of breeding populations of *I. scapularis* inhabiting forested areas within the northeast section of the state. The most common rodents in these areas were *Peromyscus* spp. mice and *M. gapperi* voles. The dominant tick species were *I. scapularis* and the American dog tick, *Dermacentor variabilis*. In that survey, a greater proportion of *M. gapperi* harbored larval *I. scapularis* ticks (10 of 23 voles) compared to *Peromyscus* mice (7 of 29) but the average number of larval *I. scapularis* per parasitized *M. gapperi* (2 per vole) was significantly less than that of *Peromyscus* (5 per mouse). In 2012, *B. burgdorferi* sensu stricto (s.s.) spirochetes (M3 strain) were cultured from the heart of an adult *M. gapperi* collected from the area (Stone et al., 2015), indicating that *M. gapperi* supports *B. burgdorferi* s.s. growth. Furthermore, *M. gapperi* has been shown to maintain *B. burgdorferi* s.l. spirochetes (MM1 strain) for up to 28 days in kidney and spleen after being injected intraperitoneally with cultured spirochetes (Bey et al., 1995). However, whether ticks can become infected with spirochetes after feeding on infected *M. gapperi* remains unknown. Thus, accumulated evidence strongly suggests that *M. gapperi* could serve as an alternative reservoir for Lyme disease spirochetes in regions where the classic reservoir species, *P. leucopus*, is absent or in low abundance. This study describes the results of field surveys in northeast North Dakota and laboratory transmission studies with locally collected *M. gapperi* and a local *B. burgdorferi* s.s. isolate to better understand the contribution that *M. gapperi* has in maintaining Lyme disease spirochetes along the northwestern edge of the vector’s current geographic range.

## 2. Materials and methods

### 2.1. Field studies

Two forested study sites in Grand Forks County, North Dakota, USA were sampled once a week for small mammals and their associated tick fauna: Turtle River State Park (47° 56′ 15″ N, 97° 30′ 17″ W), and Forest River Biology Station and Wildlife Management Area (48° 9′ 7″ N, 97° 38′ 41″ W). The sites consist of closed canopy hardwood forest encompassing ca. 250 and 350 ha., respectively, surrounded by agricultural fields. Live trapping was conducted during May through August 2012 and May through July 2013.

August 2012 and May through July 2013. At each site, approximately 25 Sherman live traps (H.B. Sherman Traps, Inc., Tallahassee, FL, USA) were baited with peanut butter and set out in the evening in a grid-like pattern. Traps were collected early the next morning. Captured rodents were anesthetized with isoflurane. Many of the captive rodents were easily identified to species level. But accurately differentiating *P. leucopus* and *P. maniculatus* in the field is unreliable (Bruseo et al., 1999; Machtinger et al., 2020). Thus, all captured *Peromyscus* mice were identified collectively as *Peromyscus*. spp. (Brown et al., 2023). Attached ticks were removed with forceps and placed in labeled vials containing 70% ethanol. After recovery from anesthesia, rodents were released at the site of capture. Sampling was conducted during May through August 2012 and May through July 2013. Ticks were returned to the laboratory, counted, and identified to life stage and species (Clifford et al., 1961; Durden and Keirans, 1996). Engorged larval ticks pulled from host animals were sorted by species and the *I. scapularis* larvae collected off each host were pooled. To test for xenopositivity of *B. burgdorferi* s.l. in the rodent population during 2012, select pools of *I. scapularis* larvae were chosen – i.e., pools from rodents that were infested only with larvae (=no co-infesting nymphs). This eliminated the possibility of detecting positive larval pools that had acquired spirochetes via co-feeding with spirochete-infected nymphs. Larval ticks were then bisected longitudinally with sterile, disposable small gauge syringe needles and the DNA was extracted using guanidine thiocyanate (Tkach and Pawlowski, 1999). The DNA extracts were then screened for the presence of *B. burgdorferi* s.l. DNA by polymerase chain reaction (PCR) using published primers and sequencing protocols as described in Russart et al. (2014).

### 2.2. Experimental co-infestation of Peromyscus with larval ticks

Studies were conducted to test whether there were differences in the anti-tick response of *P. leucopus* mice against ectoparasitism by larval *I. scapularis* versus larval *D. variabilis* ticks. A breeding colony of *P. leucopus* was established from mice captured in Grand Forks, North Dakota and pathogen-free progeny were used in experiments (see Supplementary Information). Mice were anesthetized with an intraperitoneal injection of pentobarbital (Sigma-Aldrich, Saint Louis, MO, USA) at a dose of 60 mg/kg body weight. While anesthetized, mice were placed on white filter paper on a pre-warmed slide warmer. Fur around the nape of the neck and base of the ears was shaved with electric clippers (Wahl Clipper Corp., Sterling, IL USA) and a total of forty larval ticks – 20 *I. scapularis* and 20 *D. variabilis* – were applied to each mouse by gently dabbing several ticks at a time to the ears and shaved neck with a fine-tip artist brush (#2 to 4). Mice were then loosely rolled in a strip of white paper towel and placed in a wire bottom cage suspended over a tray of water. The paper wrapping kept the mice warm while recovering from anesthesia, prevented ticks from falling off prematurely, and provided bedding for the mice during the infestation period. Trays of water were inspected daily for the next week for engorged, detached ticks. Ticks were identified and counted. A total of 16 mice were infested once. Three of the 16 mice were re-infested a second time at 14 weeks after their first tick exposure. Three of the 16 mice were exposed a total of three times at 2-week intervals.

### 2.3. Experimental transmission studies with Myodes gapperi

Studies were conducted to test the ability of *M. gapperi* voles, experimentally infected with *Borrelia burgdorferi* s.s., to infect larval *Ixodes scapularis* ticks. A breeding colony of voles was established from voles captured at the Forest River Biological Station (see Supplementary Information). After weaning, F1 offspring were maintained for 2 to 3 weeks prior to use in transmission studies.

A local isolate of *B. burgdorferi* s.s. (*OspC* group B) was cultured from heart tissue of an adult female *M. gapperi* captured in 2012 at the Forest River Biological Station site (i.e., isolate M3, see Stone et al., 2015 for details on molecular characterization). This isolate was used to infect the F1 voles. A small needle (27 gauge, 1.5 cm) was used to inject 0.1 cc of culture media containing approximately 10^7^ spirochetes subcutaneously into the occipital skin fold of each of five F1 voles. At day 10, 20 and 40 post injection (PI), voles were experimentally infested with larval *I. scapularis* ticks (obtained from the tick rearing facility at Oklahoma State University, Stillwater, OK, USA). To do this, voles were lightly anesthetized using isoflurane and placed individually into small plastic ventilated cages (approximately 3.5 × 12.5 cm). Approximately 40 to 100 larval *I. scapularis* ticks were emptied from their containment vials onto the head, nose and back of anesthetized voles. Voles were confined to the restraint cages and wrapped loosely in paper towel for the next 3 to 8 h to facilitate successful tick attachment. The restraint cages were placed inside large pans to guard against the escape of larval ticks that did not attach. Afterwards, each vole was released from its confinement and placed individually into a standard size mouse cage that had been modified such that the solid bottom had been cut out and replaced with galvanized screen with a 1.5 cm mesh. The wire-bottom cage was then suspended inside a larger, standard rat cage that contained two to three inches of slightly soapy water. This facilitated the recovery of engorged larvae that detached and dropped off. Voles were monitored daily for tick drop-off which typically occurred 4 to 6 days after attachment.

Engorged larvae were collected from the water, blotted dry, and allowed to molt to nymphs inside 25 ml plastic screw-top tubes modified such that there was a screened ventilation hole in the cap. Tubes containing potentially infected ticks were placed within sealed plastic bags containing a moist paper towel. Sealed bags were then placed within sealed plasticware containers and placed inside standard rat cages lined along the top with petroleum grease. These bio-containment procedures were done to prevent escape of potentially infective ticks. Ticks were maintained at 15 °C at high humidity for up to 5 months.

To determine how long infected voles remained infective to larval *I. scapularis*, a series of tick infestations were conducted. A total of 5 voles were infested with larval ticks 10 days after being injected with *B. burgdorferi* s.s. spirochetes. Two of the five voles were infested again on day 20 and day 40. A 40-day time frame encompasses roughly half of a typical seasonal peak abundance for larval *I. scapularis* observed in field at northerly latitudes greater than 44°N (Ogden et al., 2005, 2018; Dumas et al., 2022). After engorged larvae had detached and molted to nymphs, representative subsamples were assayed for infection – 20 nymphs from each of 5 voles after the 1st infestation, 4 nymphs from each of the 2 voles after the 2nd infestation, and 14 and 17 nymphs from the 2 voles after the 3rd infestation. For handling and sorting, nymphs were emptied out onto a chill table and macerated individually in plastic microfuge tubes using disposable plastic pestles. The DNA was extracted with a commercial kit (Qiagen, Germantown, MD, USA) and assayed for the presence of *B. burgdorferi* s.s. DNA by PCR using the primers and techniques as described in Stone et al. (2015).

Sixty-four nymphs generated from the 2 multiply infested voles (i.e., 43 nymphs from the day 20 infestation and 22 nymphs from the day 40 infestation) were kept alive for transmission studies in order to determine their ability to transmit spirochetes to naïve hosts. Using the methods described above, a total of 18 uninfected BALB/C mice were exposed to these nymphs; 9 mice were exposed to nymphs from the day 20 infestation (3 to 7 nymphs per mouse) and 9 mice were exposed to nymphs from the day 40 infestation (1 to 3 nymphs per mouse). After nymphs detached from the mice, the infection status of the ticks was assessed by assaying each tick for the presence of *B. burgdorferi* s.s. DNA using real-time PCR (Stone et al., 2015). In this way, the number of infective tick exposures was quantified for each mouse. To assess transmission success, the mice were euthanized 7 days after tick drop-off and various tissues, including tibiotarsal joint, ear, heart, and bladder, were excised and placed in BSK-II medium. The same batch of BSK-II media was used for all cultures. To ensure the quality of the BSK-II medium used for these studies, aliquots of BSK-II medium prepared ahead of time were frozen and a pre-trial aliquot was tested using *B. burgdorferi* s.s. B31 and A3 clones. The resulting spirochete growth curves were compared with our previous growth curves for these strains to ensure that the media used for these studies was good and would promote spirochete growth. Beginning at 4 to 7 days, cultures were examined daily for spirochetes using dark field microscopy. Cultures were maintained for up to 6 weeks before a sample was scored as negative (Casselli et al., 2021).

### 2.4. Data analysis

Fisher exact tests and Chi square tests were used to compare the prevalence of ectoparasitism between rodent species. Count data for ticks on infested rodents were not normally distributed for the two tick species or life stages (Shapiro-Wilk tests, *W* ≥ 0.52, *p* < 0.0001). Therefore, average tick burdens per rodent are expressed as geometric means. Kruskal-Wallis nonparametric analysis of variance with untransformed count data and *t*-tests on log_10_ transformed count data were used to determine significant differences among means. Data sorting and analyses were conducted using Microsoft Excel (Microsoft Corp., Redmond, WA USA) and Statistix 10 (Analytical Software, Tallahassee, FL USA) with the 0.05 level of significance throughout.

### 2.5. Regulatory and ethical compliance

Research involving the use of vertebrate animals was conducted in compliance with University of North Dakota Institutional Animal Care and Use Committee (UND-IACUC) approved protocols and in compliance with the Animal Welfare Act, Public Health Service Policy, and other federal statutes and regulations relating to animals and experiments involving animals. This facility where this research was conducted is accredited by the Association for Assessment and Accreditation of Laboratory Animal Care and adheres to the principles stated in the *Guide for the Care and Use of Laboratory Animals*, National Research Council, 2011. The UND IACUC specifically approved these studies.

## 3. Results

### 3.1. Field studies – small mammal composition

**Fig. 2.**
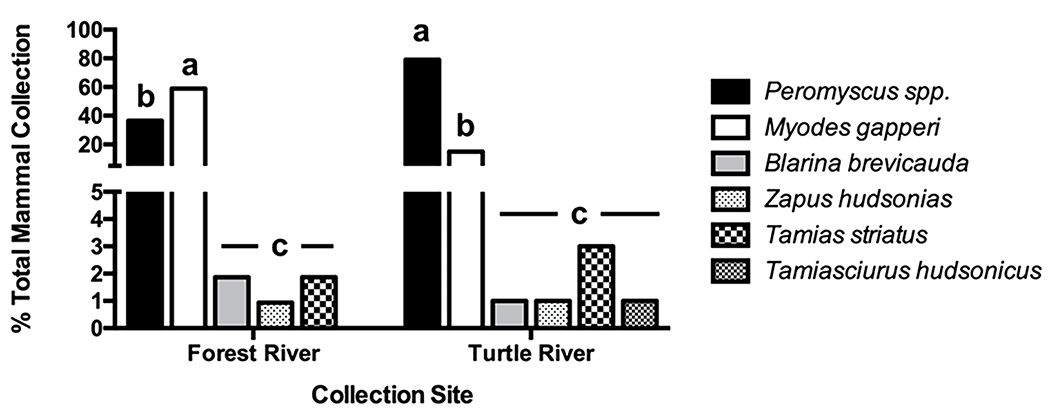
Species composition of small mammals trapped at Forest River Biological Station (*n* = 107) and Turtle River State Park (*n* = 100), Grand Forks County, ND, USA, 2012 and 2013. Within each collection site, histograms having the same letter do not differ significantly from one another at the 0.05 level.

Most (94%) of the 207 small mammals (6 species) collected consisted of *Peromyscus*. spp. (57%) and *M. gapperi* (37%). The proportions of these two rodent species differed between sites (Fig. 2). At the Turtle River site, *Peromyscus* comprised the greatest proportion (79% = 79/100) of small mammal species collected during 2012 and 2013; the proportion of *M. gapperi* collected (15% = 15/100) was significantly less (Fisher exact test, *p* < 0.00001). The reverse was true at the Forest River site where the proportion of *M. gapperi* (59% = 63/107) was significantly greater than that of *Peromyscus* (36% = 39/107) (Fisher’s exact test, *p* < 0.00001). The ratio of *Peromyscus* to *Myodes* at the Forest River site was similar in 2012 (i.e., 0.7 to 1) and 2013 (0.5 to1) (Fisher exact test, *p* = 0.33). At the Turtle River site, the ratio of *Peromyscus* to *M. gapperi* during 2012 (i.e., 8.4 to 1) was significantly higher than in 2013 (2.5 to 1) (Fisher exact test, *p* = 0.03) but nevertheless remained significantly higher than that observed at the Forest River site for both years (Fisher exact tests, p-values < 0.01). Four other mammal species were captured including the eastern chipmunk (*Tamias striatus*), meadow jumping mouse (*Zapus hudsonius*), American red squirrel (*Tamiasciurus hudsonicus*), and northern short-tailed shrew (*Blarina brevicauda*). However, these species represented collectively only ca. 5% of the total catch and therefore were not considered in subsequent analyses.

### 3.2. Field studies – prevalence of tick ectoparasitism

**Fig. 3.**
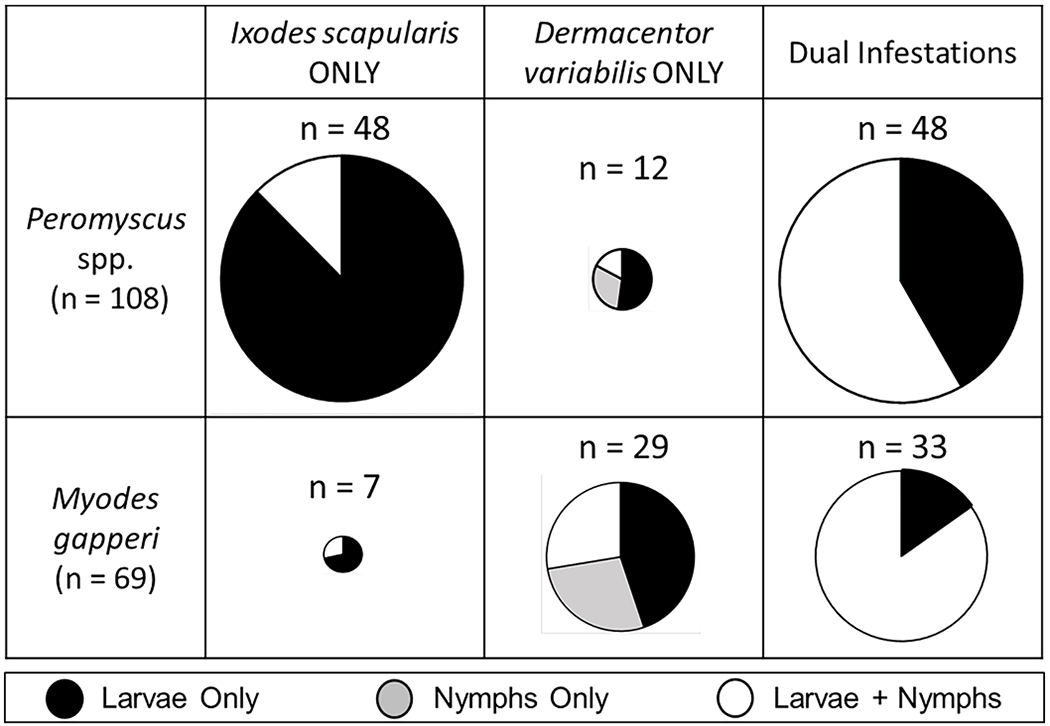
Summary of tick infestations on *Peromyscus* spp. and *Myodes gapperi* in Grand Forks County, North Dakota USA, where rodents were parasitized with either a single tick species or two tick species concurrently (=dual infestation). Pie charts show the proportion of tick life stages; where black represents larval infestations, gray represents nymphal infestations, and white represents infestations of both larvae and nymphs. The size of a pie chart is relative to the numbers of rodents collected in that category. Data are for both sites (Forest River Biological Station and Turtle River State Park) and years (2012 and 2013) combined.

A total of 1853 ticks were collected from *Peromyscus* and *M. gapperi*. Two tick species were collected – 806 *I. scapularis* (= 43% of the total) comprised of 738 larvae (40%) and 68 nymphs (4%), and 1047 *D. variabilis* (= 57% of the total) comprised of 838 larvae (45%) and 209 nymphs (11%). The overall prevalence of ectoparasitism with *I. scapularis* larvae was significantly higher for *Peromyscus* (81%) than for *M. gapperi* (47%) (Table 1; χ^2^ = 24.77, *p* < 0.0001). This was true at both sites (p-values < 0.03). There was no difference between *Peromyscus* and *M. gapperi* in overall prevalence of ectoparasitism with nymphal *I. scapularis* (20% and 21%, respectively). Conversely, the overall ectoparasitism prevalence with *D. variabilis* larvae was significantly higher in *M. gapperi* (62%) than *Peromyscus* (42%) (Table 1; χ^2^ = 6.90, *p* < 0.01). In addition, the overall ectoparasitism prevalence with nymphal *D. variabilis* was significantly higher in *M. gapperi* (47%) than in *Peromyscus* (18%) (Table 1; χ^2^ = 19.80, *p* < 0.0001). This was true for both *D. variabilis* life stages at both sites (p-values <0.05). Nearly half of tick infested *Peromyscus* (48 of 108 mice) and *M. gapperi* (33 of 69 voles) were parasitized with both tick species concurrently (i.e., “dual infestations”) and most dual infestations consisted of both larval and nymphal ticks (Fig. 3). Attached *I. scapularis* nymphs were only collected from rodents that were also parasitized with larval ticks. There was no difference in the rates of dual infestations between *Peromyscus*. spp. (44%) and *M. gapperi* (48%) (χ^2^ = 0.08, *p* = 0.77).

### 3.3. Field studies – intensity of tick ectoparasitism

Intensity of ectoparasitism was expressed as the number of ticks per infested rodent. For both sites and years combined, intensity of ectoparasitism by *I. scapularis* larvae was higher for *Peromyscus* (3.7 larvae per infested mouse) than for *M. gapperi* (2.6 larvae per infested vole), although the difference was not quite statistically significant (Table 1, F_1, 131_ = 3.7, *p* = 0.06). For both rodent species, infestation intensity by *I. scapularis* larvae was significantly higher in 2012 than 2013 (F_1, 131_ = 7.4, *p* < 0.01) and at the Forest River site versus the Turtle River site (F_1, 131_ = 3.7, *p* = 0.05). *Ixodes scapularis* nymphs were less numerous on rodents than *I. scapularis* larvae and the intensity of nymphal *I. scapularis* ectoparasitism did not differ significantly among rodent species, years, or sites (Table 1, F stats <0.46, *p* > 0.50). In contrast to *I. scapularis* larvae, the overall intensity of ectoparasitism by *D. variabilis* larvae was significantly higher for *M. gapperi* (6.0 larvae per infested vole) than for *Peromyscus* (3.3 larvae per infested mouse) (Table 1, F_1, 96_ = 5.8, *p* = 0.02). For both rodent species, the intensity of *D. variabilis* larval infestation was significantly higher at the Forest River site than the Turtle River site (F_1, 96_ = 14.8, *p* < 0.001), but there was no overall effect of year (F_1, 96_ = 0.04, *p* = 0.84). For *D. variabilis* nymphs, infestations were significantly more intense on *M. gapperi* (3.0 nymphs per infested vole) than on *Peromyscus* (1.3 nymphs per infested mouse) (Table 1, F_1, 55_ = 12.1, *p* = 0.001). As with *D. variabilis* larvae, the infestation of rodents with *D. variabilis* nymphs was significantly more intense at the Forest River site than the Turtle River site (F_1, 55_ = 5.5, *p* = 0.02) but there was no effect of year (F_1, 55_ = 1.0, *p* = 0.31).

### 3.4. Field studies – phenology of larval tick ectoparasitism

**Fig 4.**
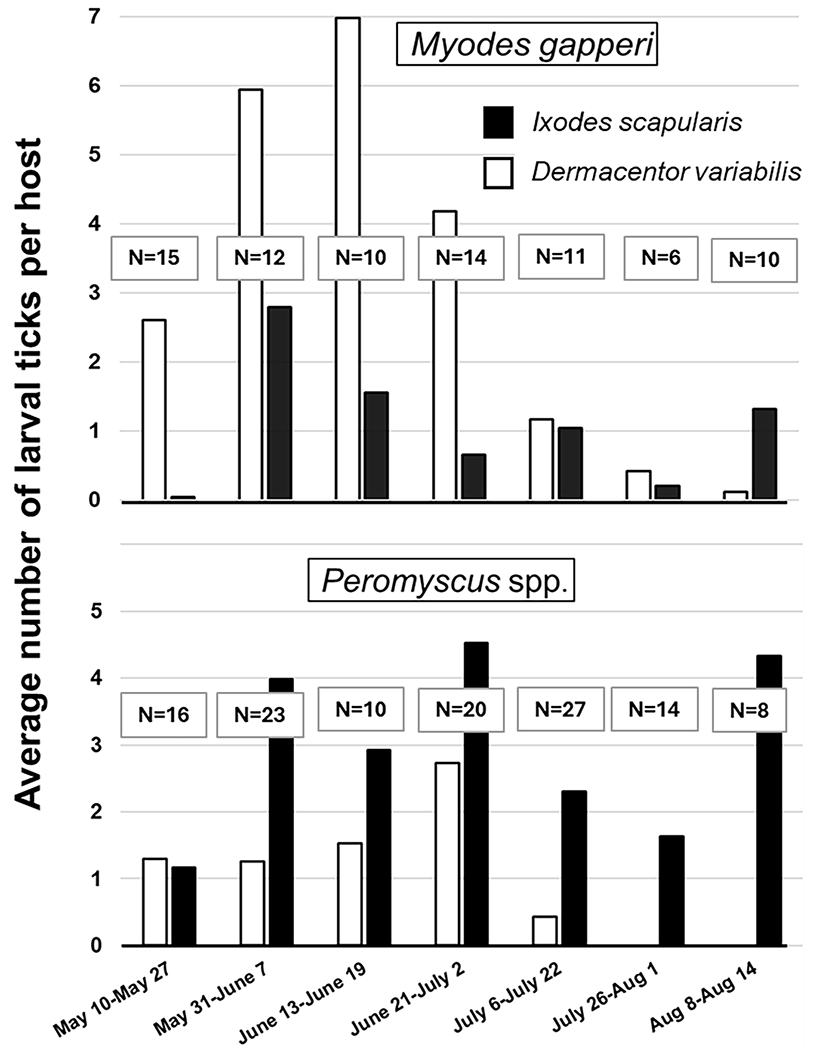
Phenology of larval tick ectoparasitism on *Myodes gapperi* and *Peromyscus* spp. rodents, where *N* = number of rodents examined. Data are for both sites and years combined, Grand Forks County, North Dakota, USA, 2012–2013.

Phenology of larval tick ectoparasitism was assessed by plotting the average number of larval ticks parasitizing rodents (including uninfested rodents) at 2-week intervals over the course of the sampling period, which roughly corresponded with trapping sessions. Ectoparasitism by *I. scapularis* larvae displayed a bimodal peak for both rodents, one in early June and another in early August (Fig. 4, black bars). Ectoparasitism by *D. variabilis* larvae displayed a single peak, with peak ectoparasitism of *M. gapperi* (mid-June) occurring earlier in the season than peak ectoparasitism of *Peromyscus*. spp. (late June) (Fig. 4, white bars). The overall level of ectoparasitism with *D. variabilis* larvae was higher for *M. gapperi* whereas the overall level of ectoparasitism with *I. scapularis* larvae was higher for *Peromyscus*. spp. Too few nymphs of either tick species were collected to construct meaningful phenology plots. Both rodent species were present throughout the sampling period with no marked peaks or crashes in populations. For *M. gapperi*, the highest capture percentage occurred in May (19% = 15 of 78 total captured) and the lowest capture occurred in late June (8% = 6 0f 78 total captured). For *Peromyscus*, the highest capture percentage occurred in July (23% = 27 of 118 total captured) and the lowest capture occurred in August (7% = 8 of 118 total captured).

### 3.5. Field studies – xenodiagnoses

The prevalence of rodents that were xenopositive for *B. burgdorferi* s.l., based on infection in attached *I. scapularis* larvae, was low (6% of 86 rodents tested), with no significant difference between *Peromyscus* and *M. gapperi* (Fisher exact test, *p* = 1.0). There was no significant difference between the Forest River (FR) and Turtle River (TR) sites in the prevalence of xenopositive rodents (TR = 10%, FR = 3%; Fisher exact test, *p* = 0.23).

### 3.6. Experimental co-infestation of Peromyscus leucopus with larval ticks

**Fig. 5.**
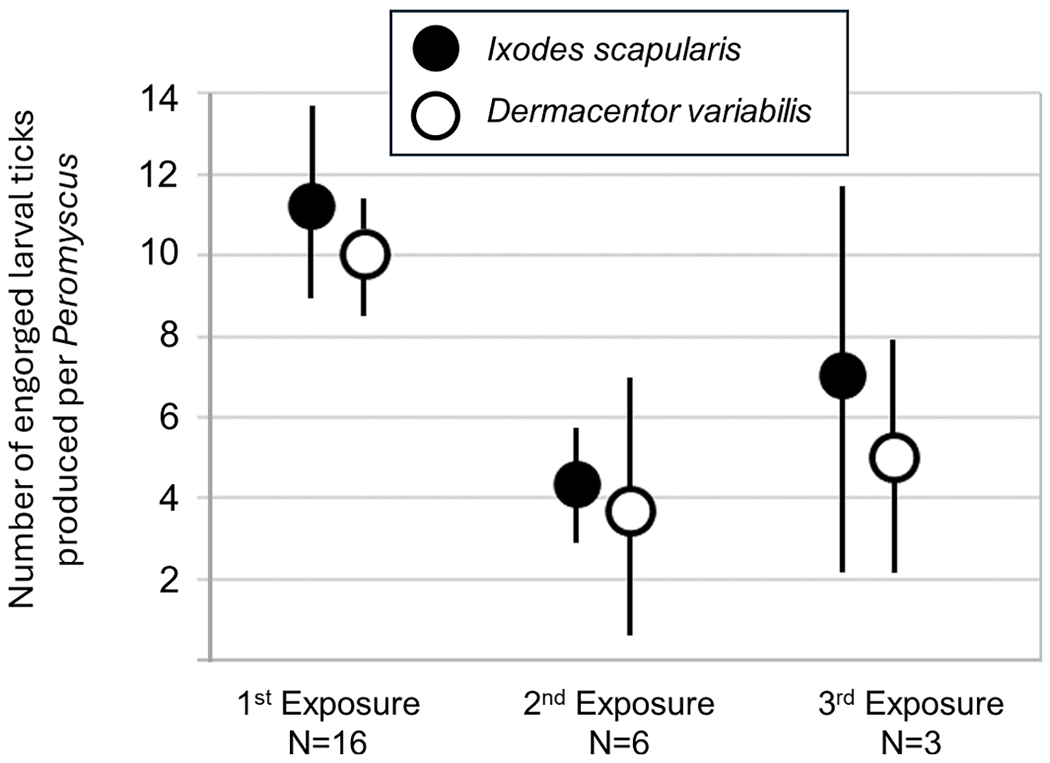
Average number of engorged larval ticks produced per mouse following experimental co-infestation of *Peromyscus leucopus* mice with 20 *Ixodes scapularis* larvae and 20 *Dermacentor variabilis* larvae. Error bars signify 95% confidence limits, *N*=number of *P. leucopus* co-infested.

Upon first exposure of 16 laboratory-raised *P. leucopus* to experimental co-infestation with 20 *I. scapularis* and 20 *D. variabilis* larvae, there was no difference between the two tick species in the average number of successful feedings that resulted (Fig. 5, paired *t*-test *t* = −0.77, df = 15, *p* = 0.45). An average of 11.1 ± 5.2 *I. scapularis* larvae and 10.0 ± 3.4 *D. variabilis* larvae engorged and dropped off the mice. Likewise, there was no difference between the two tick species in numbers of successful feedings upon a second co-infestation of six mice (paired *t*-test, *t* = −0.55, df = 5, *p* = 0.60) or upon a third co-infestation of three mice (paired *t*-test, *t* = −1.15, df = 2, *p* = 0.37). However, there were significant differences for both tick species in the average number of successful feedings that resulted between the first and second co-infestation. For the 6 mice that were infested twice, the average number of *I. scapularis* that successfully engorged on the first infestation was 13.6 ± 3.0 larvae but only 4.3 ± 1.9 larvae on the second infestation (paired *t*-test, *t* = 6.53, df = 5, *p* = 0.001). Similarly, for *D. variabilis*, an average of 9.5 ± 4.6 larvae successfully engorged on the first infestation, but only 3.7 ± 3.7 larvae engorged on the second infestation (paired *t*-test, *t* = 3.80, df = 5, *p* = 0.01). Thus, the anti-tick defense was equally effective against both tick species (Fig 5).

### 3.7. Host infectivity of Myodes gapperi – experimental transmission studies

Of the larval *I. scapularis* that fed on five experimentally infected *M. gapperi*, 61% (85/139) acquired and maintained spirochete infections through the molt to the nymphal stage (Table 2). Although there was some variation in the proportion of nymphs infected depending on the individual vole fed on and the chronicity of host infection (e.g., days 10, 20, 40 PI), the overall amount of variation was not statistically significant (F_2, 137_ = 4.39, *p* = 0.08, Table 2). Indeed, the two voles used to feed cohorts of larval ticks on days 10, 20, and 40 PI (i.e., V2 and V8) generated remarkably similar proportions of infected nymphs on days 10 and 40 PI (60 to 65%, Table 2).

Infected nymphs successfully transmitted *B. burgdorferi* s.s. to 72% of 18 BALB/c mice (Table 3). There was no significant difference in the transmission rates between the nymphs generated from larvae infected on day 20 PI (78% transmission rate) versus those generated from larvae infected on day 40 PI (67% transmission rate) (Fisher exact test, *p* = 0.18). Most (66%) of the 64 engorged nymphs recovered from the laboratory mice tested positive for *B. burgdorferi* and all 18 laboratory mice were exposed to at least one infected tick (Table 3). However, spirochete transmission was variable and unpredictable. For example, three laboratory mice became infected after being bitten by a single infected tick (mice VII, X, XIII) whereas three other mice did not (mice IX, XVII, XVIII). Two laboratory mice remained uninfected despite being fed upon by multiple infected ticks (*e.g.*, mice VIII, XVI). Overall, three of six mice (50%) became infected after exposure to a single infected tick bite whereas 10 of 12 mice (83%) became infected after exposure to two or more infected tick bites (Table 3). However, this difference was not significant (Fisher exact test, *p* = 0.17), perhaps due to low sample size. Diagnosis of transmission relied on culturing of live spirochetes from laboratory mice one week after nymphs had detached. To do this, four anatomically distinct tissues – urinary bladder, tibio-tarsal joint, heart, and ear – were taken from each laboratory mouse. Tissue type had a significant effect on successfully culturing and detecting spirochetes (χ^2^ = 10.44, *p* < 0.05). Spirochetes were detected in a significantly higher proportion of laboratory mice when examining cultures derived from bladder (100%) and leg joint tissue (77%) compared to ear (46%) and heart (31%) tissues χ^2^ tests, p-values < 0.05, Table 3).

### 3.8. Reservoir potential of Peromyscus. spp. versus Myodes gapperi

The relative contributions of *Peromyscus*. spp. versus *M. gapperi* in maintaining Lyme disease spirochetes at the Forest River and Turtle River sites (i.e., comparative reservoir potentials) were determined based on the model of Mather et al. (1989) (Table 4). This model considers estimates of rodent abundance, *I. scapularis* larval tick burden, and reservoir infectivity of each rodent species for *B. burgdorferi* s.l. The value for reservoir infectivity of *M. gapperi* was determined in our laboratory as described above whereas the value for *Peromyscus* was taken from published literature (Donahue et al.,1987). At Turtle River State Park, *Peromyscus* was more abundant and supported higher prevalence and intensity of larval *I. scapularis* ectoparasitism than did *M. gapperi*. Consequently, *Peromyscus*. spp. was responsible for nearly all (0.93) of the enzootic maintenance of Lyme disease spirochetes at that site while *M. gapperi* contributed very little (0.06) (Table 4). However, at the Forest River site less than 50 km away, the estimated reservoir potentials for *Peromyscus* (0.57) and *M. gapperi* (0.43) were more comparable. The most influential components in determining differences in reservoir potential were site-specific differences in relative rodent abundance and a higher overall level of larval *I. scapularis* ectoparasitism on *Peromyscus* versus *M. gapperi*.

## 4. Discussion

The geographic distribution of *I. scapularis* and the pathogens this tick transmits (e.g., Lyme disease spirochetes) have expanded considerably in recent decades and northeastern North Dakota currently represents the northwestern-most limit for this tick species within the central USA (Eisen and Eisen, 2023). This region is typified by large expanses of industrial-scale agriculture and suitable habitat for *I. scapularis* (i.e., closed-canopy forest) is confined to fragmented patches, mostly along riverine corridors. Our study examined the two largest forest fragments within Grand Forks County, ND in order to characterize the enzootic transmission system for spirochetes in this region. Over 90% of the small mammal fauna trapped within these disjunct forests consisted of *Peromyscus* mice and *M. gapperi* voles. *Peromyscus* was the dominant small mammal species at the Turtle River site (79% of the total) whereas *M. gapperi* dominated the Forest River site (58% of the total). At both sites, the rodents were parasitized by the immature stages of two tick species: *D. variabilis* (historically endemic to the region; Bishopp and Trembley, 1945) and *I. scapularis* (recently introduced; Russart et al., 2014). Concurrent infestation of individual rodents by both tick species was common (Fig. 3). *Borrelia burgdorferi* s.l. was circulating among the ticks and rodents at both sites as determined by xenodiagnoses of engorged larval *I. scapularis* removed from both *Peromyscus* and *M. gapperi*.

To understand the relative roles that *Peromyscus* and *M. gapperi* have as reservoirs for *B. burgdorferi* s.l. at each of these sites, we calculated site-specific reservoir potentials for both species (Table 4). To do this required empirical estimates of the abilities for each rodent to infect larval *I. scapularis* with *B. burgdorferi* s.s. spirochetes (i.e., host infectivity). The ability of *Peromyscus* mice to become infected and successfully transmit *B. burgdorferi* s.l. to *Ixodes* ticks is well established (Donohue et al., 1987; Rand et al., 1993; Peavy and Lane, 1995) but similar information for *M. gapperi* was not available and had to be determined experimentally. In laboratory studies, we found that the host infectivity of *M. gapperi* was essentially equivalent to that of *P. leucopus* (Tables 2 and 3). This was not altogether surprising since it had been determined previously that *M. gapperi* was capable of developing spirochetemia after inoculation with *B. burgdorferi* s.l. (Bey et al., 1995). However, our experimental results confirmed definitively that *M. gapperi* can serve as a reservoir for Lyme disease spirochetes.

Defining host infectivity is essential to the process of reservoir incrimination but it does not, by itself, define the relative importance of a reservoir species. Our field studies demonstrated that the relative importance of *M. gapperi* as a reservoir also depended on its abundance and the degree to which it was parasitized by immature *I. scapularis* ticks. For example, at the Turtle River site, *M. gapperi* was 5 times less abundant than *Peromyscus* and contributed very little to the overall reservoir potential at that site (6%, Table 4). Conversely at the Forest River site, the abundance of *M. gapperi* exceeded that of *Peromyscus* by almost two-fold. Yet the estimated reservoir potential of *M. gapperi* (43%) was still less than that of *Peromyscus* (57%). Because host infectivity of the rodents was equivalent, the difference was attributed to an asymmetry of host utilization by tick larvae at these sites. Ectoparasitism by *I. scapularis* larvae was consistently higher on *Peromyscus* than on *M. gapperi* at both sites while the reverse was true for *D. variabilis* larvae (Table 1). Similar results have been recorded in tick surveys where sympatric populations of *Peromyscus* and *M. gapperi* have been examined for ticks (see Table 5). In each of these surveys, immature *I. scapularis* particularly larvae, were more strongly associated with *Peromyscus* than with *M. gapperi* (Table 5). Unfortunately, the strong association of immature *D. variabilis* ticks with *M. gapperi* observed in our survey was not corroborated by these surveys because *D. variabilis* ectoparasitism was extremely low, absent, or not recorded. Nevertheless, these studies support our observation that when *Peromyscus* mice and *M. gapperi* voles live together in areas that contain breeding populations of *I. scapulars* ticks, *Peromyscus* will accrue more *I. scapulars* larvae than *M. gapperi* and, all else being equal, *Peromyscus* will have a higher reservoir potential.

Interestingly, similar asymmetries of host utilization by larval ticks have been observed in the closely related *Ixodes ricinus-B. burgdorferi* s.l. system in northern Europe. Studies in France (Matuschka et al., 1999; Perez et al., 2017), the Netherlands (Gassner et al. 2013), and Norway (Mysterud et al., 2019) have reported significantly higher ectoparasitism by *I. ricinus* larvae on wood mice (*Apodemus sylvaticus*) than on sympatric bank voles (*Myodes glareolus*).

The apparent asymmetry of host utilization by larval ticks observed in our study sites was critical in estimating reservoir potentials for the two dominant rodent species inhabiting the sites. Yet the potential reason(s) for these asymmetries are not fully understood. Here, we propose two hypothetical mechanisms which are not necessarily mutually exclusive – 1) differences in host anti-tick defenses (either immunological or behavioral [e.g., grooming]) – i.e., the “tick resistance hypothesis”, and 2) differences in host encounters with larval ticks in the environment – i.e., the “spatial ecology hypothesis”.

In the tick resistance hypothesis, foraging *Peromyscus* mice and *M. gapperi* voles may encounter clusters of larval ticks more or less equally but *Peromyscus* would exhibit a reduced immunological and/or grooming response to *I. scapularis* larval tick bites (Anderson et al., 2017; Cull et al., 2017) compared to the anti-tick response of *M. gapperi*. But for this hypothesis to fully explain our field results, *Peromyscus* should also exhibit a more vigorous anti-tick defenses against *D. variabilis* larvae – a so-called dichotomous immune response (see Narasimha et al., 2021). Such a dichotomous immune response in *Peromyscus* exposed to repeated co-infestations with larval *I. scapularis* and *D. variabilis* ticks was not observed in our experimental trial (Fig. 5) and is contrary to the tick resistance hypothesis. However, it should be pointed out that the anti-tick response of *M. gapperi* was not evaluated in this study, nor was the response of *Peromyscus* and *M. gapperi* to larval infestations of each tick species individually. There is still much to learn about anti-tick responses in different host species against different larval tick species. Although our co-infestation experiment (Fig. 5) does not support the tick resistance hypothesis, it is premature to discount the role that anti-tick responses may have played in the asymmetry of host utilization by larval *I. scapularis* and *D. variabilis*.

An alternative scenario, i.e., the spatial ecology hypothesis, suggests that differences in foraging habits among different reservoir species may determine the likelihood of encountering clusters of larval ticks within the environment (Shaw et al., 2003). That is, the microdistribution of *Peromyscus* mice foraging within the study sites may have been more closely aligned with that of *I. scapularis* larvae whereas the microdistribution of *M. gapperi* aligned more closely with that of *D. variabilis* larvae. With respect to microdistribution patterns of the rodents, several ecological studies found no strong interspecific competition for space or resources between sympatric populations of *Peromyscus* and *M. gapperi* (Miller and Getz, 1977; Wolff and Dueser, 1986; McCracken et al., 1999; Wood et al., 2017). This suggests that these rodents exhibit niche partitioning. *Peromyscus* mice are considered semi-arboreal generalists (Lackey et al., 1985; Klein et al., 2012), likely to forage for insects, seeds, and fruit on the surface of the forest floor, along tops of fallen logs, etc. In contrast, *M. gapperi* voles are considered semi-fossorial specialists (Merritt, 1981), likely to forage deeper in the forest litter in search of fungus and lichens, which are major constituents of their diet (Orrock et al., 2002; Stephens et al., 2020; Tisell et al., 2023).

With respect to microdistribution of the larval ticks, it is generally assumed that the place where engorged female ticks detach from their host determines the habitat where eggs are laid and hatch (Leal et al., 2020). Hatchling ticks typically form clusters. It has been observed that I. *scapularis* larvae do not disperse widely from where egg masses have been deposited (Daniels and Fish, 1990; Stafford, 1992). Thus, the location of aggregated clusters of larval ticks is most likely determined by where blood-engorged female ticks drop off their hosts (Leal et al., 2020). Adult *D. variabilis* mostly parasitize medium-sized mammals (e.g., canines, racoon, skunk) (Zimmerman et al., 1988; Kollars et al., 1993, 2000) whereas adult *I. scapularis* mostly parasitize deer (Spielman et al., 1979). If a substantial proportion of engorged female ticks detach during the time when their reproductive hosts are at rest, then it is likely that host resting sites are sites where clusters of larval ticks will be most common.

The reproductive hosts for *D. variabilis* – i.e., mesocarnivores (e.g., skunks, raccoons, opossums) – are nocturnally active during the tick season but typically rest during the day in hidden retreats (*e.g.*, dens, logs, etc.) (Storm, 1972; Shirer and Fitch, 1970; Rabinowitz and Pelton, 1986; Lariviere and Messier, 1997). Diurnal refuges (sometime referred to as ‘day-beds’) are seldom used on consecutive days (Shirer and Fitch, 1970; Lariviere and Messier, 1997). If engorged *D. variabilis* ticks drop off while their hosts rest within refuges, then it is likely that after a period of time, clusters of *D. variabilis* larvae will appear in these types of sites and likely to be encountered by foraging, semi-fossorial *M. gapperi* voles. The reproductive host for *I. scapularis* – i.e., deer – do not seek dens or refuges but rather rest out in the open. In this case, it is likely that clusters of *I. scapularis* larvae may be more abundant in the upper layers of the forest litter and more likely to be encountered by *Peromyscus* mice foraging on the surface of the forest floor. The spatial differences in rodent foraging habits and larval tick microdistribution may have contributed to the asymmetry of host utilization observed at our study sites.

Data on the spatial distribution of tick egg masses and dispersal of larval ticks within nature remain scant (Leal et al., 2020) and clearly more information is needed before it can be determined whether the spatial ecology hypothesis fully accounts for why one reservoir species harbored more larval ticks than another. Even so, our studies suggest there was substantial overlap in rodent-specific encounters with questing larvae because a large proportion of tick-infested *Peromyscus* (44%) and *M. gapperi* (48%) were concurrently infested with larvae of both tick species (Fig. 3, see also Butler et al., 2021).

In conclusion, we found that the southern red-backed vole, *M. gapperi*, is a suitable reservoir host and can contribute to the enzootic cycle of Lyme disease spirochetes in northeastern North Dakota and possibly other areas as well, such as central Canada (Boonstra and Krebs, 2012), Appalachian Mountains (Wood et al., 2017), and coastal Maine (Elias et al., 2022), where this rodent species is a major component of the small mammal fauna. The observation that reservoir potentials for two sympatric rodent species can vary widely within a geographic range of less than 50 km underscores the importance of estimating the relative abundance of host species within a local habitat and quantifying their respective contributions to feeding and infecting larval ticks (Kilpatrick et al., 2017; Halsey et al., 2018; Goethert and Telford, 2022).

## Supplementary Material

1

2

## Figures and Tables

**Table 1:** Prevalence (%) and intensity (geometric mean and 95% confidence limits of ticks per infested rodent) of larval and nymphal ticks parasitizing *Peromyscus* mice and *Myodes gapperi* voles at two forested sites in Grand Forks County, North Dakota, USA, in 2012 and 2013.

Site / Year	Rodent	N	*Ixodes scapularis*				*Dermacentor variabilis*			
			Prevalence		Intensity		Prevalence		Intensity	
			Larvae	Nymphs	Larvae	Nymphs	Larvae	Nymphs	Larvae	Nymphs
Forest River 2012	*Peromyscus*. spp.	26	92%	27%	3.3 (2.0, 5.1)	1.3 (0.7, 2.4)	65%	31%	3.8 (2.2, 6.5)	1.2 (0.9, 1.8)
	*Myodes gapperi*	38	66%	18%	3.1 (2.0, 4.5)	1.7 (0.9, 3.3)	63%	61%	6.6 (4.0, 11.0)	3.8 (2.5, 5.8)
Forest River 2013	*Peromyscus*. spp.	13	77%	31%	2.8 (1.4, 5.6)	1.3 (0.5, 3.1)	62%	23%	4.9 (1.9, 12.5)	1.8 (0.5, 7.2)
	*Myodes gapperi*	25	16%	20%	1.6 (0.7, 3.7)	1.4 (0.8, 2.7)	64%	32%	9.1 (5.4, 15.6)	2.2 (1.0, 5.0)
Turtle River 2012	*Peromyscus*. spp.	59	95%	34%	4.7 (3.8, 6.1)	1.4 (0.9, 2.3)	31%	15%	3.3 (2.2, 4.8)	1.4 (0.9, 2.0)
	*Myodes gapperi*	7	100%	29%	1.9 (1.1, 3.4)	1.0 (nd)*	57%	57%	2.2 (0.2, 28.9)	2.4 (0.4, 14.7)
Turtle River 2013	*Peromyscus*. spp.	20	30%	15%	1.2 (0.7, 1.9)	1.3 (0.5, 3.4)	35%	5%	1.7 (0.9, 3.7)	1.0 (nd)*
	*Myodes gapperi*	8	13%	25%	5.0 (nd)*	1.4 (nd)*	50%	25%	1.5 (0.7, 3.7)	1.0 (nd)*
Total	*Peromyscus*. spp.	118	81%	20%	3.7 (3.1, 4.6)	1.3 (1.1, 1.7)	42%	18%	3.3 (2.5, 4.4)	1.3 (1.1, 1.7)
	*Myodes gapperi*	78	47%	21%	2.6 (2.0, 3.6)	1.5 (1.1, 2.0)	62%	47%	6.0 (4.2, 8.5)	3.0 (2.1, 4.1)

* nd = not determined. Confidence limit could not be computed due to low sample sizes (≤ 2).

**Table 2:** Infectivity of F1 *Myodes gapperi* voles to larval *Ixodes scapularis* ticks, where voles were inoculated by needle with *Borrelia burgdorferi* sensu stricto spirochetes and cohorts of larval ticks fed on voles at 10, 20, and 40 days afterwards. Engorged larval ticks molted to nymphs and were then tested for *B. burgdorferi* s.s. infection status.

Incubation period of *B. burgdorferi* s.s. in voles at time of larval tick feeding (days)	Vole ID	Number of nymphs tested	Number of positive nymphs	Infection rate	Mean ± SD
10	V2	20	12	60.0%	59.0 ± 6.5%
	V5	20	13	65.0%	
	V6	20	10	50.0%	
	V7	20	11	55.0%	
	V8	20	13	65.0%	
20	V2	4	3	75.0%	75.0 ± 0%
	V8	4	3	75.0%	
40	V2	14	9	64.3%	64.5 ± 0.3%
	V8	17	11	64.7%	
TOTAL		139	85		61.2 ± 1.3%

**Table 3:** Recovery of *Borrelia burgdorferi* sensu stricto spirochetes from various tissues harvested from BALB/c *Mus musculus* mice one week after infected *Ixodes scapularis* nymphs had engorged and detached. Nymphs were infected as larvae by feeding on *Myodes gapperi* voles at either 20 days or 40 days after voles were given a single subcutaneous injection of a North Dakota isolate of *Borrelia burgdorferi* s.s.

Incubation period of infected voles	Mouse ID	No. nymphs engorged	No. nymphs infected (%)	Transmission outcome	Tissues with culturable spirochetes			
					Bladder	Joint	Ear	Heart
20 days	I	4	4 (100%)	Infected	+	+	+	
	II	6	5 (83%)	Infected	+	+	+	
	III	5	4 (80%)	Infected	+	+		
	IV	7	4 (57%)	Infected	+	+	+	
	V	4	2 (50%)	Infected	+	+		
	VI	5	2 (40%)	Infected	+			+
	VII	3	1 (33%)	Infected	+	+	+	
	VIII	4	3 (75%)	Not Infected				
	IX	4	1 (20%)	Not Infected				
40 days	X	1	1 (100%)	Infected	+	+	+	+
	XI	3	3 (100%)	Infected	+	+		
	XII	3	3 (100%)	Infected	+			+
	XIII	1	1 (100%)	Infected	+	+		
	XIV	3	2 (67%)	Infected	+	+	+	
	XV	3	2 (67%)	Infected	+			+
	XVI	2	2 (100%)	Not Infected				
	XVII	2	1 (50%)	Not Infected				
	XVIII	3	1 (33%)	Not Infected				
Overall Infection Rates (N)			66% (64)	72% (18)	100%	77%	46%	31%
					—— out of 13 total infected mice ——			

**Table 4:** Estimated reservoir potentials of *Peromyscus* spp. and *Myodes gapperi* to maintain enzootic transmission of *Borrelia burgdorferi* sensu lato at two noncontiguous forested tracts within Grand Forks County, North Dakota, USA, in 2012 and 2013.

Site	Rodent species	Relative abundance^a^	*Ixodes scapularis* larvae per rodent^b^	Host infectivity^c^	Per capita production of infected nymphs^d^	Relative reservoir potential
Forest	*Peromyscus*	1.606	2.694	0.642	1.730	0.566
River	*Myodes*	2.739	1.270	0.612	0.810	0.434
Turtle	*Peromyscus*	3.810	3.286	0.642	2.083	0.935
River	*Myodes*	0.762	1.186	0.612	0.757	0.065

a Average number of rodents captured per sampling session.

b Proportion of hosts parasitized by larval ticks (=prevalence) multiplied by the number of larval ticks per parasitized host. (=intensity). See Table 1 for prevalence and geometric mean intensity values.

c Probability of larval *Ixodes scapularis* ticks acquiring an infectious dose of spirochetes and metamorphosing into infective nymphs after engorging on a spirochete-infected host (see Table 2 [*M. gapperi*] and Donohue et al., 1987 [*Peromyscus*]).

d Number of larval ticks per rodent multiplied by specific infectivity. This assumes 100% post-engorgement survival and successful molting of detached larvae.

**Table 5:** Prevalence of ectoparasitism by immature *Ixodes scapularis* and *Dermacentor variabilis* ticks among sympatric populations of *Peromyscus* mice and *Myodes gapperi* voles within North America.

Location	Years	Tick Life Stages	Tick Species	*Peromyscus*	*Myodes gapperi*	Citation
North Dakota, USA	2012 & 2013	Larvae	*Ixodes scapularis*	81.4% (118)	47.4% (78)	Present Study
			*Dermacentor variabilis*	42.4% (118)	61.5% (78)	
Maine, USA	1989 to 2019	Larvae	*Ixodes scapularis*	13.6% (5,551)	0.6% (1,566)	Elias et al., 2022
			*Dermacentor variabilis*	0.2% (5,551)	0.2% (1,566)	
Quebec, Canada	2007 & 2008	Larvae	*Ixodes scapularis*	13.7% (1,363)	3.0% (168)	Bouchard et al., 2011
			*Dermacentor variabilis*	0% (1,363)	0% (168)	
Wisconsin, USA	2012 to 2014	Larvae + Nymphs	*Ixodes scapularis*	42.5% (576)	0% (123)	Larson et al., 2018
			*Dermacentor variabilis*	Ticks present but data not reported		
Pennsylvania, USA	2018 & 2019	Larvae + Nymphs	*Ixodes scapularis*	79.6% (470)	25.8% (291)	Brown et al., 2023
			*Dermacentor variabilis*	0% (470)	0% (291)	

## Data Availability

Data are provided as an excel file in the Supplementary Materials.
