# Supplementary material for: The role of southern red-backed voles, Myodes gapperi, and Peromyscus mice in the enzootic maintenance of Lyme disease spirochetes in North Dakota, USA
Source: Ticks Tick Borne Dis. Author manuscript; Available in PMC 2024 Dec 26. (PMC11670892; doi:10.1016/j.ttbdis.2024.102385)
Supplement: 1 [file NIHMS2042337-supplement-1.docx]

**Supplementary Material**

Field-captured rodents were placed in quarantine for 10 to 14 days during which time they were examined and treated for ectoparasites, intestinal parasites, and screened for prior exposure to hantavirus. Only rodents that were free of parasites and sero-negative for hantavirus were allowed into the breeding programs. Field-collected rodents were housed individually in standard rat cages for several days to acclimate to captivity. Rodents were provided cardboard tubes to hide in and were maintained on a diet of rodent pellets supplemented with succulent greens (e.g., celery, grasses). To treat for ectoparasites (fleas, mites, ticks), voles were dusted with 5% carbaryl insecticide (Sevin®; Central Garden & Pet Co., Walnut Creek, CA, USA) and *Peromyscus* were sprayed with a mixture of 0.1% permethrin, 0.05% pyrethrins, 0.5% piperonyl butoxide (DuMOR Equine Fly Spray®, Farnum Co. Inc., Phoenix, AZ, USA). To treat for intestinal helminths, rodents were treated via oral gavage with 2 mg moxidectin per kilogram body weight and 125 mg praziquantel per kilogram body weight (QuestPlus Gel® horse de-wormer; Zoetis, Parsipanny, NJ, USA). Two to three days after treatment, rodents were examined for evidence of intestinal parasites *via* microscopic examination of their feces, specifically looking for helminth eggs and protozoa. Rodents were tested for prior exposure to hantavirus (*i.e*., anti-hantavirus IgG) by direct enzyme-linked immunosorbent assay (ELISA) using the following protocol. The capture antigen was recombinant protein of the nucleocapsid from Sin Nombre virus obtained from BEI Resources (Catalog No. NR-9670; BEI Resources, Manassas, VA, USA). The ELISAs were started by adding a 50 ml of a capture antigen (0.025 µg/ml) in phosphate-buffered saline (PBS) solution to each well of 96-well microtiter plate (Corning® Costar®, Corning, NY, USA). Plates were covered and incubated overnight at 4°C. Wells were emptied and incubated for one hour at room temperature with 0.5% boiled casein blocking buffer. Boiled casein was prepared by suspending 5 grams casein in 100 ml 0.1 N NaOH and bringing the solution to a boil. After the casein dissolved, 900 ml of PBS was added, and the solution was allowed to cool. The pH was adjusted to 7.4 with hydrochloric acid (1 M HCL) and 0.1 grams of thimerosal plus 0.2 grams of phenol red were added. Samples and commercial reagents were diluted in 0.5% boiled casein; 50µl per well volumes were used throughout, and plates were covered during incubations. Assays were conducted at room temperature. Test sera from field-collected *Peromyscus* and *M. gapperi* were diluted serially 1:200 to 1:1,000. In addition to test sera, each plate included positive and negative control wells. Positive and negative control rodent sera were diluted serially 1:1000 to 1:32,000 and tested. Positive control serum was high-tittered polyclonal antiserum to the nucleocapsid protein of Sin Nombre virus produced by immunization of deer mice (*Peromyscus maniculatus* Wagner) with the recombinant Sin Nombre nucleocapsid protein (Catalog No. NR-9676; BEI Resources, Manassas, VA, USA). Negative control sera were collected from non-immune laboratory mice (*Mus musculus*). After an hour incubation at room temperature, wells were emptied and washed 3 times with PBS plus 0.05% Tween 20. The detection system consisted of affinity-purified polyclonal antibody to whole *P. leucopus* IgG, H+L chains, made in goat, and labeled with horseradish peroxidase (SeraCare, Milford, MA, USA). This was diluted 1:4000 in boiled casein buffer and incubated for 1 hr. Wells were emptied and washed 6 times with PBS plus 0.05% Tween 20 and the enzyme substrate added (SureBlue TMB 1-Component Microwell Peroxidase Substrate; SeraCare, Milford, MA, USA). Absorbance values (414nm) were recorded after 30 minutes using an ELISA plate reader. Samples were tested in triplicate and the mean and standard deviation (SD) calculated for each dilution. The cutoff value determining the ELISA endpoint titer of a sample was defined as the mean absorbance value of the appropriate negative control + 3 SD.

After treatments were complete and rodents had cleared quarantine, breeding pairs were housed together for seven days after which the male was removed. Females gave birth approximately 10 days later. Three weeks after birth, offspring (F1) were removed from their mother and housed individually. Offspring (mixed sexes) were maintained for another 2 to 3 weeks prior to use in experimental studies.
